# Photonic Integrated Circuit Based Temperature Sensor for Out-of-Autoclave Composite Parts Production Monitoring

**DOI:** 10.3390/s23187765

**Published:** 2023-09-08

**Authors:** Georgios Syriopoulos, Ioannis Poulopoulos, Charalampos Zervos, Evrydiki Kyriazi, Aggelos Poulimenos, Michal Szaj, Jeroen Missinne, Geert van Steenberge, Hercules Avramopoulos

**Affiliations:** 1 Photonics Communications Research Laboratory, National Technical University of Athens, 9 Iroon Polytechniou Street, Zografou, 15773 Athens, Greece; 2 Engineering Technology Solutions E.E., 15344 Athens, Greece; 3 Argotech a.s., Holubova 978, CZ-547 01 Náchod, Czech Republic; 4 Center for Microsystem Technology (CMST), Ghent University and IMEC, Technologiepark 126, 9052 Ghent, Belgium

**Keywords:** temperature sensor, Bragg gratings, optical, PIC, composites, photonics, production monitoring

## Abstract

The use of composite materials has seen widespread adoption in modern aerospace industry. This has been facilitated due to their favourable mechanical characteristics, namely, low weight and high stiffness and strength. For broader implementation of those materials though, the out-of-autoclave production processes have to be optimized, to allow for higher reliability of the parts produced as well as cost reduction and improved production speed. This optimization can be achieved by monitoring and controlling resin filling and curing cycles. Photonic Integrated Circuits (PICs), and, in particular, Silicon Photonics, owing to their fast response, small size, ability to operate at higher temperatures, immunity to electromagnetic interference, and compatibility with CMOS fabrication techniques, can offer sensing solutions fulfilling the requirements for composite material production using carbon fibres. In this paper, we demonstrate a passive optical temperature sensor, based on a 220 nm height Silicon-on-Insulator platform, embedded in a composite tool used for producing RTM-6 composite parts of high quality (for use in the aerospace industry). The design methodology of the photonic circuit as well as the experimental results and comparison with the industry standard thermocouples during a thermal cycling of the tool are presented. The optical sensor exhibits high sensitivity (85 pm/°C), high linearity (R^2^ = 0.944), and is compatible with the RTM-6 production process, operating up to 180 °C.

## 1. Introduction

Composite materials have seen increased use in the aerospace industry, due to their high strength–weight ratio, high thermal stability, and design flexibility. These characteristics have justified them as a replacement of metal alloys in aircraft and automobile structural components. The repercussions of those replacements, and also the potential for recyclability and fuel economy, also drive research in the automotive industry, in the face of ambitious climate accords [1].

The highest quality parts are produced using an autoclave, with tightly controlled temperature, pressure, and vacuum conditions, and multiple heating and cooling cycles to cure the resin [2]. Even using smarter procedures to reduce the number of cycles, the massive upfront investments and the excessive energy consumption of those processes, make the overall costs prohibitive for use cases demanding aerospace quality composite parts. For this, out of autoclave processes have also seen adoption, which use metallic and composite tools (moulds) to manufacture composite parts, with lower pressure conditions. The greatest trade-off of this method lies in the quality of the parts produced. For the required quality of the composite parts, and faster curing cycles, tighter monitoring and control of the production process is required [3].

To achieve this, a variety of monitoring solutions have been proposed, with the most prominent being thermocouples, capacitive sensors, and fibre Bragg grating sensors. The electrical solutions suffer in terms of size, both for the wiring and the sensors themselves, making it difficult to be embedded in composite tools. More importantly, their susceptibility to electromagnetic interference as well as their limited range makes their use in carbon fibre composite tools prohibitive [4]. Photonic structures have a strong track record in sensing applications [5,6,7,8], and there are configurations of sensing optical fibres deployed in composite parts for structural health monitoring [9,10,11]. However, these sensors are often embedded at a certain depth in the tool or the produced part, thus prohibiting monitoring at the interface between the tool and the resin. 

**Figure 1:**
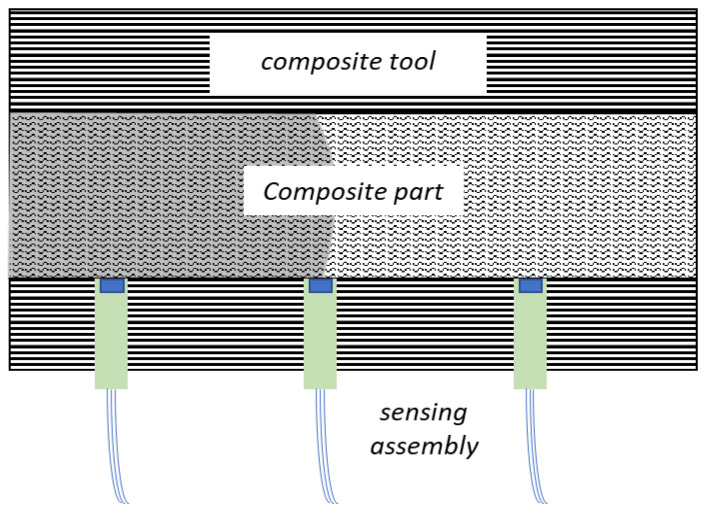
Schematic of the overall concept, with the sensor at the resin–tool interface.

Therefore, the solution that we propose in this manuscript is the use of a Photonic Integrated Circuit (PIC) sensor seamlessly embedded into the production tool. Employing Bragg grating sensors on a PIC, the advantages of these type of sensor structures known from their implementation in fibres are still in effect, namely, their electromagnetic interference immunity [12], the miniaturization potential [13], and the ability to multiplex signals, while the photonic structures can be placed right at the surface of the production tool, thus allowing better monitoring of the resin’s curing cycles, while having a minimal “imprint” risk on the surface of the composite part. This concept is demonstrated in Figure 1, as the miniaturized sensors are embedded in the composite tool and deployed in close proximity with the heated resin.

The main performance criteria for temperature sensing usually include sensitivity, range, accuracy, response time, and power efficiency [14]. For the silicon photonics configuration proposed, the response time is rapid, and the optical losses for wavelengths used are minimal. The rest of the requirements are decided by the specific composite production process being monitored. The work presented in this manuscript focuses on an RTM-6 process. The sensitivity that can be achieved with the proposed platform is >50 pm/°C, which, depending on the measurement setup, can lead to a resolution smaller than 0.5 °C [15]. The sensing range is well within the requirements of the RTM-6 process (<185 °C).

## 2. Concept

The applied sensor is a Bragg grating structure, based on an integrated photonics platform, consisting of a periodic corrugation of the refractive index, namely, the waveguide width [16,17]. This exact periodicity in the structure’s geometry causes a selectivity in the spectrum, acting as a band-pass filter. As a result, the sensor will reflect a certain part of the wavelength spectrum, which depends on the grating pitch but also on external conditions such as temperature. It is worth noting that the PIC is fabricated on a 220 nm Silicon-on-Insulator platform, using the ISiPP50G platform by IMEC, on a passive-only design run of a Multi-Project Wafer (MPW) service, opting for strip waveguide structures. The high refractive index contrast between the silicon and its surrounding oxide is what allows the guiding of the light through the silicon strip. The silicon structures are designed for maximum sensitivity to temperature and accuracy of measurement. Since all silicon optical structures suffer from spectral shifts, due to their thermo-optic sensitivity, the calibration of this Bragg sensor will also act as a reference to a future multi-sensor, enabling differential measurements and isolating the response due to pressure and refractive index. The top (low temperature) oxide has enough height to shield the temperature sensor from the optical interaction with the resin, and in a foreseen implementation of a refractive index sensing element, the Bragg sensing area will need to be exposed through post-processing of the low temperature oxide. The data from those sensors will enable the monitoring and control of a full RTM-6 resin injection and curing process. 

**Figure 2:**
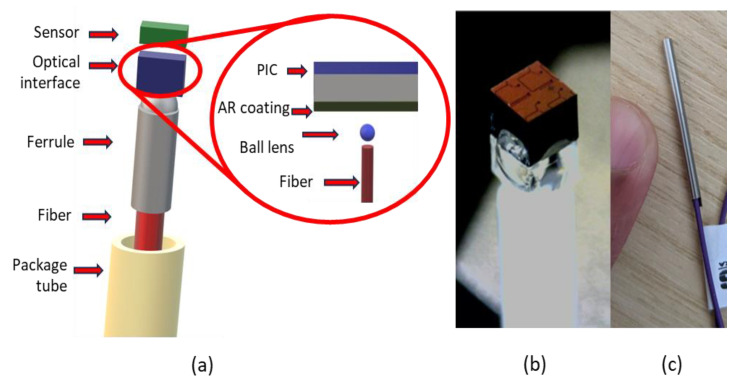
(**a**) The packaged sensor configuration (**left**), with the fibre-to-PIC interface (**right**). (**b**) The sensor build-up, with the PIC on top and the interface to an optical fibre at the bottom side. (**c**) The sensor inside the package tube, connected to an optical fibre.

Light in PICs travels in submicrometre-sized waveguides, making it challenging to couple external light (e.g., from/to a fibre) in and out of the PIC. A standard method to interface a PIC with a (single mode) fibre is to use a grating coupler [18,19]. Since the sensor should be in contact with the part to be monitored, the fibre cannot be attached to the surface of the PIC. As such, the PIC should be interfaced from the back side. Moreover, due to the PIC substrate thickness (~720 μm), additional optics are required to focus the spot from the fibre onto the grating coupler. We have developed a method based on a standard microball lens to achieve this in an efficient way, which, also, due to its curvature, collects the backscattering light from the grating coupler into the optical fibre [20]. This solution requires only polishing and (optionally) antireflective coating of the PIC back-side surface, making it suitable for small-scale optical PIC manufacturing. A 3D holder is also utilized for housing the ball lens, which is fabricated in fused silica, using a femtosecond laser for the “drawing” of the required contour and Potassium Hydroxide (KOH) etching of the irradiated parts. The fibre, holder with ball lens, and PIC are precisely mounted on top of each other using a dedicated assembly station and, in a last step, are packaged in a protective metal tube [21]. Figure 2a presents a schematic of the mechanical and optical packaging of the PIC, as well as a schematic of the fibre–chip interface. The temperature sensor can be seen in Figure 2b,c, with the optical PIC on the top side and the optical and mechanical packaging at the bottom side for a sense of scale. 

**Figure 3:**
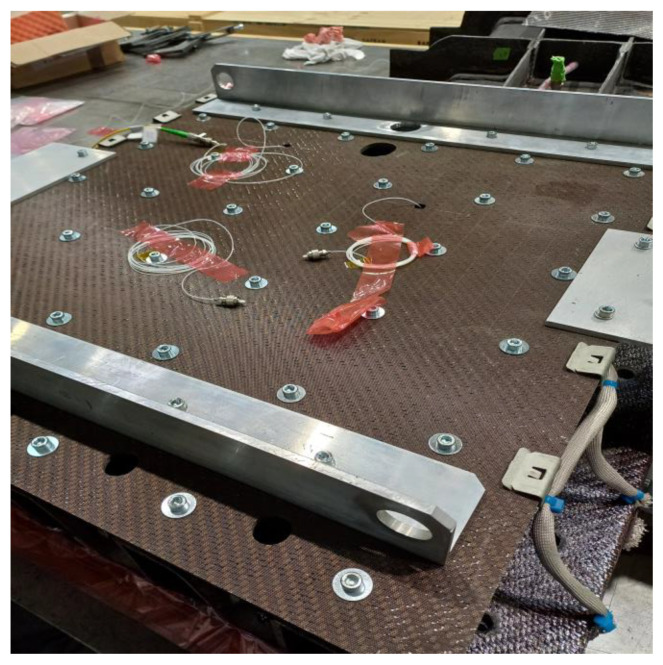
The composite tool, with the integrated sensors embedded, with their interfacing fibres visible.

The packaged sensors are embedded in the composite tool utilizing Through Thickness Reinforcement (TTR) techniques [22]. The process starts by inserting metal surrogate pins into the preform, which is the precursor material to the composite tool end-product, using quasi-static insertion into the heated preform. After the curing cycle is completed, the surrogate pins are substituted with the packaged sensors. This procedure causes minimal damage to both the tool and the sensors and does not require specialized equipment. Figure 3 shows the composite tool with integrated optical sensors, of which the interfacing fibres are visible.

## 3. Materials and Methods

### 3.1. Design of the Bragg Grating

The Bragg gratings are surface-relief structures and fabricated on a 220 nm Silicon-on-Insulator platform. A single grating pitch consists of a silicon strip with two different values of width. The materials used and their refractive indices are listed in Table 1.

It needs to be mentioned that only the optical properties of the materials are modelled, without considering the thermal expansion of the materials. This will not be a limitation though, since the small dimensions of the waveguide and the low value of the thermal expansion coefficient of silicon [23] give an expansion of the waveguide dimensions in the picometer regime. Thus, the effect of the waveguide’s thermal expansion, in the optical response of the sensors, can be safely ignored.

The design process starts with a 2-dimensional study, the calculation of all waveguided modes, and their associated effective index value ($n_{eff}$). The computational tool used is a cross-sectional algorithm, namely, FDE (Finite Difference Eigenmode), available in commercial Lumerical packages. It calculates both the effective index of its mode, as well as its spatial profile. The algorithm makes use of a Yee mesh, as in Finite Difference Time domain (FDTD) algorithms, but in contrast with them, the FDE algorithm solves the Maxwell’s equations in the frequency domain.

#### 3.1.1. Waveguide Modes Calculation

The algorithm is executed in two parts, for both sections of a single grating pitch. The core concept of the design consists of applying a corrugation width (dw) across the structure, by adding it in the width of the first section and subtracting it in the next, of each grating period. The “starting” (or “DC”) waveguide width for the perturbation of the sidewall is 450 nm, while the height of the grating remains unchanged at 215 nm throughout the structure, due to platform specifications. Thus, the only degree of freedom in a single grating pitch lies in the difference in width between the two parts. Through the calculation of the effective index of each of the guided modes ($n_{eff}$), the grating pitch (Λ) can be estimated, for achieving the required resonance wavelength ($\lambda_{B}$), based on the expression:

(1) $$\lambda_{B}=2\cdot n_{eff}\cdot \Lambda$$

We also have to mention that the effective refractive index is also affected by the ratio of the wider part, to the total grating pitch, in the propagation direction. This number is called Filling Factor (FF). As an FF of 50% is chosen, both parts of the grating pitch have an equal contribution to the $n_{eff}$.

**Figure 4:**
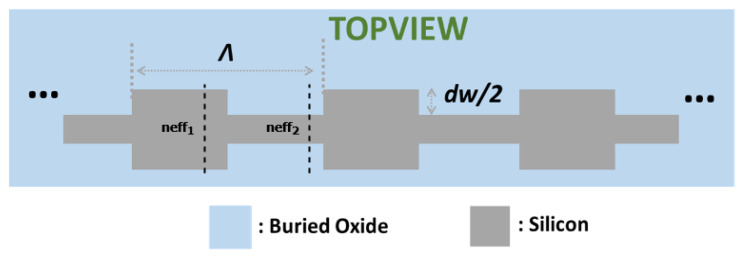
Conceptual top view of a Bragg grating showing the design variables.

Therefore, $n_{eff}$ is calculated by $n_{eff}={0.5n}_{eff1}+0.5n_{eff2}$, also shown in the Figure 4. 

For compatibility with established fibre Bragg grating technology already used in the composite industry, resonant wavelengths ($\lambda_{B}$) in the C-band are taken into consideration. With resonance at around 1550 nm, and considering the expression above, certain values for the grating pitch will correspond to TE- or TM-guided modes; therefore, structures are uniquely designed for different polarization states.

#### 3.1.2. Propagation Simulation

Following from the calculation of the grating pitch for the required resonant wavelength, the next step is to optimize the spectral response of the optical structures, to facilitate greater accuracy in the detection of the resonant wavelength in the reflection lobe. The metrics that will define the choice of dw (and the corresponding Λ) will be the peak reflectivity (the height of the reflection lobe) and the bandwidth of the lobe, measuring the 3 dB bandwidth. For faster response time, the simulations are carried out using frequency domain algorithms. For this, EME (EigenMode Expansion) propagation solver by Lumerical is utilized, which works by analysing the field into guided and radiation modes and modelling the discontinuities of the structures using scattering matrix techniques. Since the scattering matrix of each period is identical, this reduces the computational complexity significantly [24], making it a suitable choice for modelling periodic structures, while factoring in the finite length of the structure, such as the one we demonstrate.

Table 2 presents the effects of the geometrical characteristics’ variation in the optical response of the periodic structure.

The designs result in a photolithography mask, which is used for their fabrication with standard semiconductor manufacturing techniques. Upon receiving the fabricated PICs, they are diced and packaged. The photonic sensors are tested, both in a laboratory setting, as shown in [16,17], and inside a composite tool.

### 3.2. Experimental Characterization

**Figure 5:**
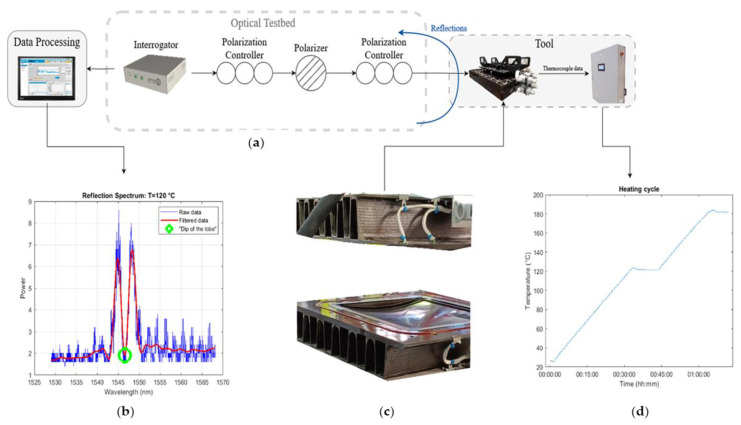
(**a**) Schematic of the experimental testbed. (**b**) The response of the optical sensor, raw data (blue) and filtered data (red), with respect to wavelength. (**c**) Matrix and punch of the composite tool. (**d**) Heating cycle, as recorded by the thermocouples.

Figure 5 shows the experimental testbed consisting of a regular computer, fibre interrogator, and the optical filtering configuration composed of the polarizer and the polarization controllers. The composite tool includes both the embedded thermocouples and the optical sensors. The response of the sensor, the filtered optical data and the centre of the dip are shown on the bottom left corner, as a function of wavelength. All the thermocouple data are directed to a control box, and the thermocouple values are compared to the response in the optical domain. On the bottom of the figure, the punch (top) and the matrix (bottom) of the composite tool is demonstrated, as well as the part of the thermocouples that is not embedded.

In detail, for testing of the photonic structures, compatibility with established fibre Bragg infrastructure is warranted. Therefore, a commercial laser interrogator (FAZT I4G) [26] is deployed, covering up to 39.2 nm in the C-band, from 1529 nm to 1568.2 nm, with TE or TM polarization, while reading the spectrum of the reflections of the Bragg structures. The rest of the optical experimental testbed consists of optical fibres, a polarizer with a 45° angle, and two polarization controllers on each side of the polarizer. This setup is deployed to filter any excess noise from the rest of the PIC and align the polarization state of the light to the polarization operation state of the sensor. The sensors are tested for vacuum integrity, validating their ability to withstand the pressure from the injection of resin for multiple cycles. The packaged PICs are also tested in multiple heating cycles, according to RTM-6 process requirements.

The sensors were embedded in a composite tool, both in the matrix and punch, which is heated on different zones due to the composites’ low thermal conductivity. The tool is tested in a dry run setting, without a resin flow, with the duration of the cycle exceeding one hour and the full curing cycle exceeding two hours, taking the cooling into account. When the temperature reaches 120 °C, it is stabilized for several minutes. The wavelength values of the centre of the reflection lobe are assigned to values of temperature by using several thermocouples in each heating zone, and the results are compared to the ones obtained from the lab measurements. 

We also have to mention that the data are processed with a Chebyshev filter of the second type, for high execution speed, therefore allowing fast monitoring of the process. After the optimization of the filter, the values for the passband and the stopband “frequency” are 0.0003 and 0.0009, respectively. The attenuation for the stopband is set to 100 dB and the allowed ripple at the passband is at 0.1 dB. It is worth a reminder that although the filter “sees” dB values of power, the interrogator does not provide exact values for the power of the Bragg grating response.

## 4. Results and Discussion

### 4.1. Design of the Bragg Grating

#### 4.1.1. Waveguide Mode Calculation

**Figure 6:**
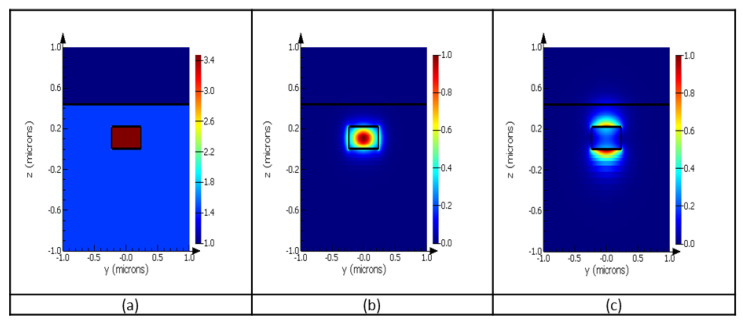
(**a**) Cross-section of the layer stack indicating the refractive indices for each material used; (**b**) the electrical field intensity profile for TE mode; (**c**) the electrical field intensity profile for TM mode, for the same silicon waveguide.

We mentioned in Section 3.1.1 that the starting waveguide width is 450 nm, which is in the range of values for single-mode operation for a wavelength of 1550 nm, while exhibiting low waveguide losses. Figure 6 presents the profile of a structure with a 450 nm width and a height of 215 nm. The field intensity of the two polarization states (TE and TM) can be seen in Figure 6b,c, as well as the refractive index of the materials utilized for the chips (a). We can observe that the electric field is more confined in the silicon core in the TE polarization than in the TM.

**Figure 7:**
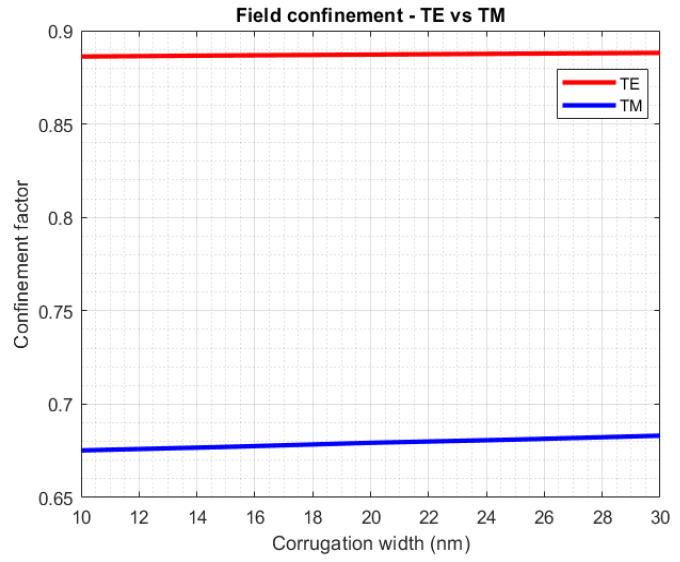
Confinement factor as a function of grating corrugation width for both TE and TM polarizations.

Figure 7 demonstrates the confinement factor (*y*-axis) for the TE polarization state (red) and the TM (blue), as a function of the corrugation width (*x*-axis). The confinement factor is the percentage of power guided through a waveguide core, normalized to the total power. The difference in the optical power distribution is evident, with higher power distribution inside the silicon strip in TE mode. Since silicon is the material with the highest thermo-optic coefficient, therefore displaying high sensitivity in temperature change, the choice was made to include only TE structures in the testing phase. 

**Figure 8:**
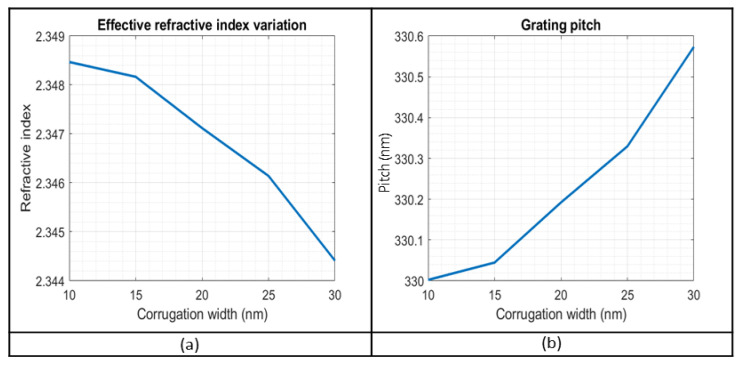
(**a**) The effective refractive index of the fundamental mode for TE polarization. (**b**) The grating pitch as a function of the corrugation width.

Figure 8a presents the effective refractive index for the fundamental mode, as a function of the corrugation width, to account for the effect of corrugation width in the $n_{eff}$, also shown in Figure 4. Through the expression $\Lambda =\lambda_{B}/2\cdot n_{eff}$, for $\lambda_{B}=1550\;\mathrm{nm}$, the corresponding grating pitch is calculated, and is presented in Figure 8b, for corrugation width within a range of 10 nm up to 30 nm.

As will be shown later, the recorded spectrum in the experimental phase has a range of 1529 nm up to 1568 nm. With a rise in temperature, the reflection spectrum moves towards higher wavelengths. So, the gratings’ response has to be skewed towards the lowest parts of the available spectrum to capture the spectrum across the temperature range. As a result, the structures that will be fabricated will have period lengths of 324, 326, and 328 nm, to keep the resonance at the required wavelength. 

#### 4.1.2. Propagation Simulation Study

**Figure 9:**
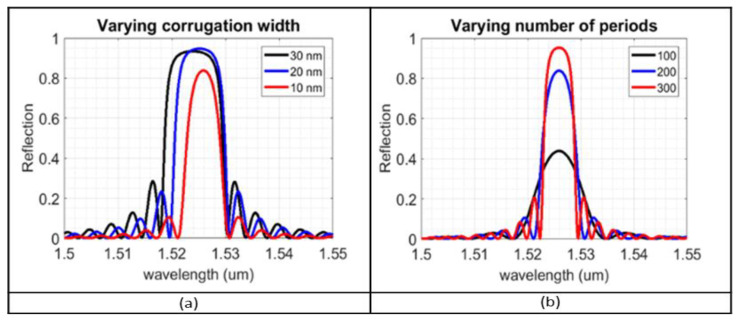
Change in reflection spectra, caused by a variation in: (**a**) corrugation width; (**b**) number of periods.

Having calculated the grating pitch for the range of values of the corrugation width, the design proceeds to the 3D modelling of the device in the optical domain, first at room temperature and following with varying temperature simulations. The variables of interest are now the corrugation width and the total number of periods. Figure 9 displays the effect on the simulation metrics, based on varying different geometric characteristics, for a Bragg structure with 10 nm, 20 nm, 30 nm corrugation width, and 100, 200, and 300 periods. The objective is to maximize the peak reflectivity and minimize the 3 dB bandwidth, for the most accurate tracking of the centre of the lobe in the experimental phase, over the entire temperature range. We can see that a corrugation width of 20 nm provides the highest peak reflectivity for 200 periods, as well as a 3 dB bandwidth of 3 nm. With an increase in the number of periods, the lobe’s bandwidth is reduced, though much longer gratings (>500 periods) are more likely to be affected by tight fabrication tolerances and defects, leading to a broader peak. 

**Figure 10:**
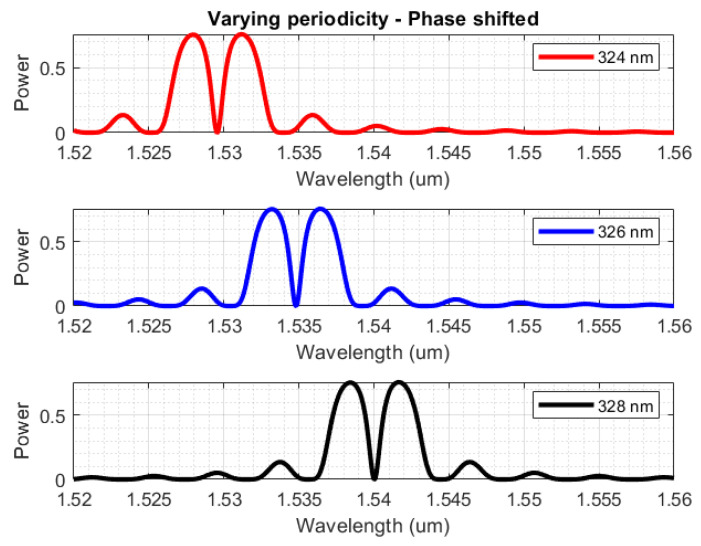
Reflection spectra for phase-shifted Bragg gratings with TE polarization, for configurations of dw = 20 nm and Λ = 324 nm, 326 nm, 328 nm.

It is worth noting that a variant of regular Bragg gratings was also investigated, specifically, gratings with an unperturbed period in the middle of a regular structure (π-phase shift), causing a strong dip in the reflection lobe. This dip makes for a far more accurate tracking of the centre of the reflection lobe, and the design process is almost identical to that of the regular Bragg gratings [27]. Figure 10 shows some indicative spectrums for various configurations of the phase-shifted Bragg grating simulations, for a structure with dw = 20 nm and Λ = 324 nm, 326 nm, 328 nm, and 400 periods (200-phase shift-200). As expected, the variation of the grating pitch affects the lobe only in its spectral position. 

**Figure 11:**
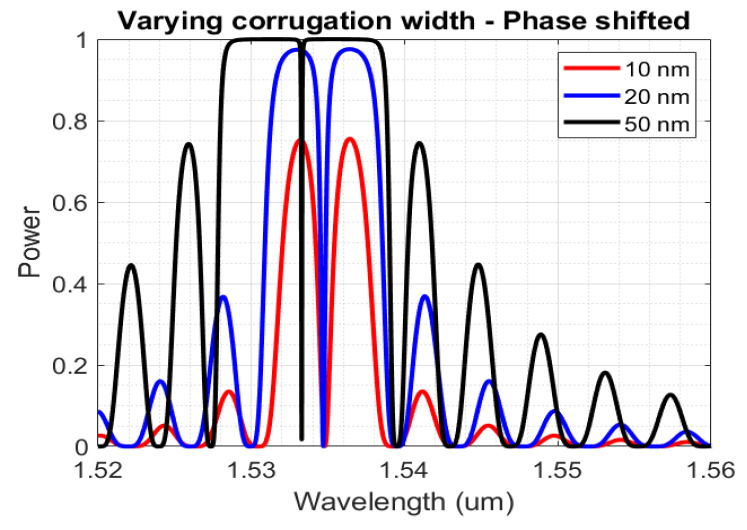
Reflection spectra for phase-shifted Bragg gratings with TE polarization, for configurations of Λ = 326 nm and dw = 10 nm, 20 nm, 30 nm.

A significant change in the optical response can be observed, as shown in Figure 11, by varying the corrugation width of the Bragg grating. With a higher dw, the dip in the spectral response has its bandwidth value decreased. At 30 nm though, the 3 dB bandwidth of the dip is less than 40 pm. Τhis value will render the tracking of the dip more precarious during the characterization, considering the significant noise in the reflection spectrum, that is associated with the interfacing of the PIC to the fibre, with the total reflection spectrum of the sensor occupying a greater part of the spectrum. Therefore, the deployed sensors will have a 20 nm corrugation width. 

**Figure 12:**
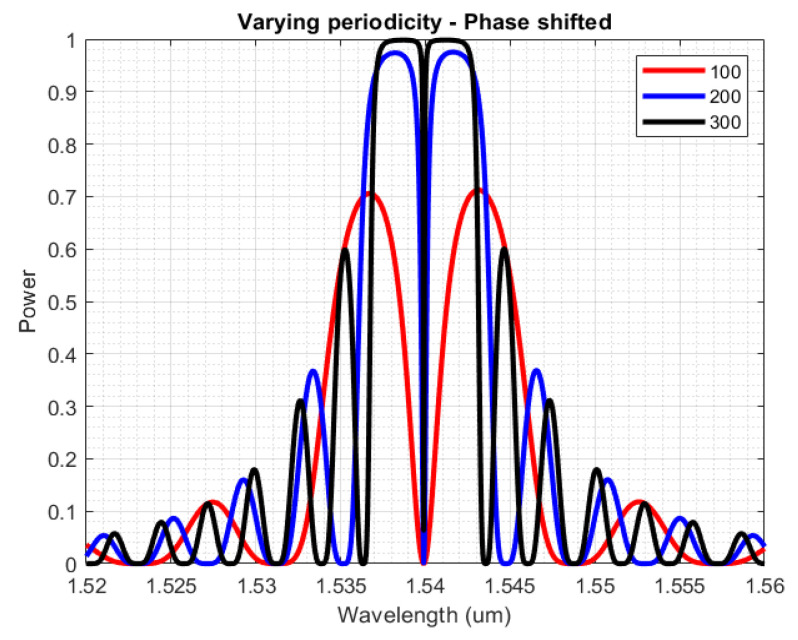
Reflection spectra for phase-shifted Bragg gratings with TE polarization, for configurations of Λ = 326 nm, dw = 20 nm, and periodicity of 100, 200, 300.

The final part of the design process, under stable temperature conditions, concerns the periodicity of the phase-shifted grating. Figure 12 presents the reflection spectrum for a phase-shifted Bragg grating, with Λ = 326 nm and dw = 20 nm, for 100 up to 300 periods. As observed in this graph as well as in Figure 9b for regular gratings, a relatively small number of periods (<200) will increase the spectral bandwidth of the optical sensors, as well as the peak reflectivity, leading to less accurate detection of the central wavelength in the experimental phase. Above 200 periods, the dip of the reflection lobe becomes significantly spectrally narrower, making it harder to detect with our experimental setup. For the reasons above, we applied the same number of periods as for regular Bragg sensors (200) on each side of the pi phase shift.

**Figure 13:**
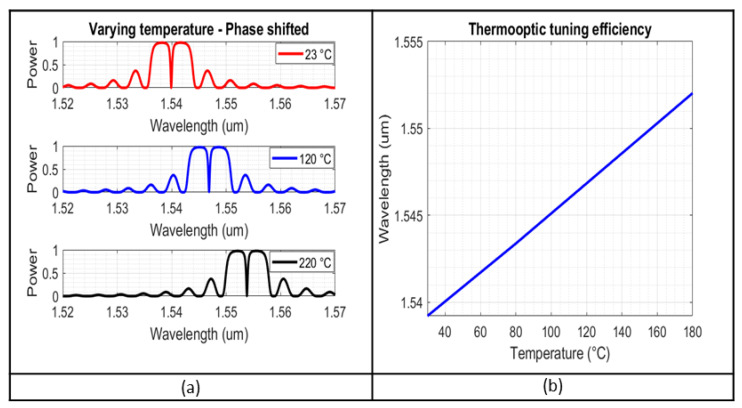
(**a**) Reflection spectra at varying temperature, for phase-shifted Bragg grating, of Λ = 326 nm, dw = 20 nm, N = 200 for temperatures of: 23 °C, 80 °C, 180 °C; (**b**) temperature sensitivity of the same structure.

After a range of values for the geometrical characteristics is selected, corresponding to the requirements for the peak reflectivity and the 3 dB bandwidth, the simulations for the temperature variation are carried out, based on the thermo-optic coefficient of silicon, of $\frac{dn}{dT}=18\times 10^{-5}\left(K^{-1}\right)$ for wavelength values around 1.55 μm [28]. Figure 13a presents indicative reflection spectra affected by the variation of the ambient temperature, for the phase-shifted gratings, with Λ = 326 nm and dw = 20 nm and 200 periods. 

The temperature sensitivity is at 85 pm/°C. The high linearity of the sensors’ behaviour will facilitate the interpretation and the further processing of the results. We conclude that the structures with the most desirable behaviour are the ones with dw = 20 nm, and 200 periods. Nonetheless, the resulting mask will include structures with geometries across the range presented in previous sections, to account for any deviations in the fabrication process.

### 4.2. Experimental Results

**Figure 14:**
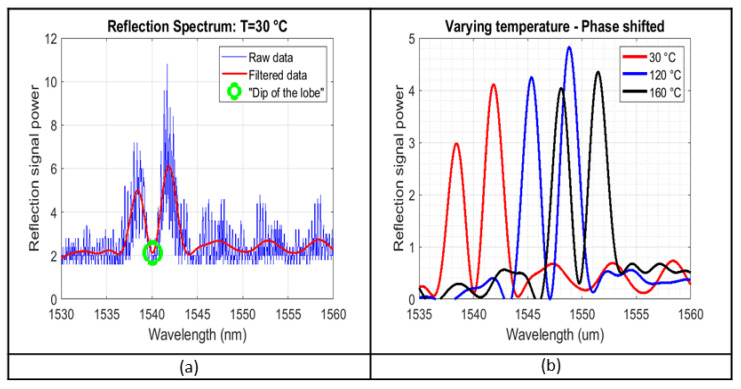
(**a**) Reflection spectra, raw data, filtered data and dip in the reflection lobe for 30 °C. (**b**) Filtered spectrum data for 3 different temperature levels.

The experiment took place in two phases. The first was the calibration of the sensors in a lab environment. As presented in [17], a sensitivity of ≈0.078 nm/°C is measured, with the response being highly linear. The second phase, involving optical sensors in the composite tool, was conducted at the premises of Loiretech Ingénierie, and as mentioned above, they were composed of heating cycles reaching up to 180 °C. Figure 14a demonstrates some indicative raw and filtered sensor reflection spectra across the range of the temperature values. The sensor signal from the Bragg was strong enough to observe the red shift of the reflection spectrum with increasing temperature, as shown in Figure 14b, which presents the response of the optical sensors (post-filtering) across the temperature range. The scale of the response time of the digital filter is in the millisecond range. 

It is worth mentioning that the y-axis of the interrogator’s spectrum analyser, presenting the reflection power, only provides a qualitative picture, and does not provide absolute values. Since just the spectral shift is monitored, though, that is not limiting. With a spectral rate of 16 Hz, the data rate is fast enough for the dwell-time of RTM-6 heating cycles, which is the time spent on certain curing temperatures (depending on the resin used).

We briefly mentioned in Section 3.1 the integrity testing as well as the heating cycles that the sensors underwent. We validated the optical sensor operating in a real environment against the optical sensor calibration performed in laboratory conditions using the calibration in lab conditions.

**Figure 15:**
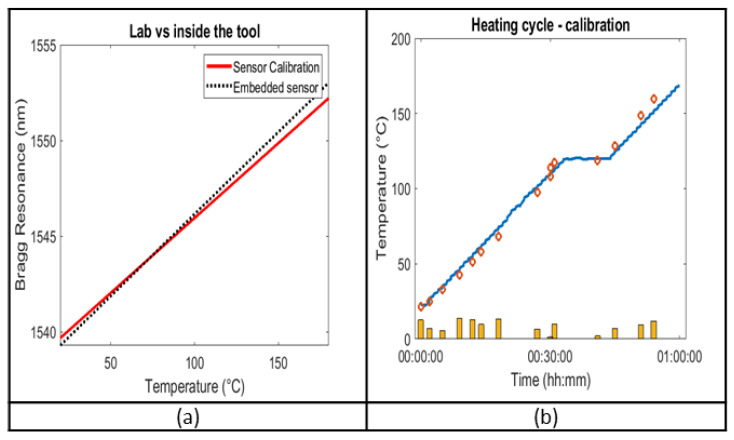
(**a**) Linear regression models for the sensor in the lab, and inside the tool. (**b**) Heating cycle, as recorded by the thermocouple (blue), and the temperature as recorded by the optical sensors (red dots). The yellow bars provide a qualitative overview of the total error.

The data from the sensors integrated inside the tool can be compared to the calibration from the initial experimental testbed in the lab, as shown in Table 3. The two measurements are in agreement, validating both the calibration and the embedding process, with the greatest similarity in response being observed between 40 °C and 100 °C. The difference is mostly an effect of a divergence in the lab calibration, though the embedding of the sensors also may cause a slight shift in the resonance wavelength. Figure 15a shows the linear regression models for the sensor response, both in a laboratory setting and embedded in a tool, with respect to temperature. For a linear regression model with coefficients a,b in y≈ax+b, please see Table 3.

The difference between the Bragg response expected from the design and the experimental validation in the lab environment, although minor, needs to be mentioned. A key reason for this inconsistency lies in errors in the experimental calibration, with the digital filtering of the data having a minor influence. 

In the fabrication of the optical structures, the highest dimensional error is in the width of the waveguide, which causes a minor detuning and a variation in the shape of the reflection lobe, as it was also noted in the design section. The steep dip of the reflection lobe, though, assures that the tracking of the reflections’ centre remains accurate for phase-shifted gratings, increasing the tolerance for manufacturing error. Variations in the waveguide height have a more pronounced effect on the reflection spectrum, though, causing a detuning. Discrepancies in the optical height are more evident in chips diced from the edges of the wafer, as measured by the optical setup presented above, reaching up to 2.5%.

The consistency between the calibration and the embedded sensor measurements allows us to advance to the in-tool validation of the sensors, using the initial calibration curve, converting the dip in the reflection spectrum to temperature values. These temperature readings are then evaluated with respect to the thermocouple data.

Figure 15b compares the response of the thermocouple closer to the Bragg grating sensor with the values of an optical sensor, with Λ = 326 nm, dw = 20 nm, N = 200, as a function of time. The results, shown above, critically include a number of errors from the experimental testbed, with the error of the K-type thermocouples being one of them, given at ±0.75% (1.35 °C at 180 °C). Another source of discrepancies lies in the difference in temperature levels recorded between the two testbeds. All of them accumulate in total errors with a maximum of ±5% with the bars at the bottom of the graph providing a qualitative representation of the percentage of the difference between the optical sensors and the thermocouples.

## 5. Conclusions

We presented the design and experimental evaluation of a silicon photonics Bragg grating sensor for monitoring key metrics in composite production processes. We demonstrated the design simulation results for regular and phase-shifted Bragg gratings, the experimental evaluation results in lab environment, as well as the experimental validation results with the sensor embedded in a composite tool. The results demonstrate a deployed composite tool, with embedded temperature sensors, showing high sensitivity (85 pm/°C) and linearity. This enables rapid measuring of temperature across the range of the RTM-6 composite production process, at the interface of the composite tool, thus allowing for monitoring of the resin’s state, with minimum sensor footprint.

## Figures and Tables

**Table 1:** The materials used in the integrated structure.

Material	Refractive Index
Silicon	3.47
Buried Oxide (BOx)	1.45
Top Oxide	1.41

**Table 2:** The geometrical characteristics and their effect in the optical response of the sensor.

Variable	Effect
Corrugation width	$\mathrm{Determines}\;\mathrm{peak}\;\mathrm{reflectivity},\;3\;\mathrm{dB}\;\mathrm{bandwidth},\;\mathrm{and}\;\mathrm{has}\;a\;\mathrm{minor}\;\mathrm{effect}\;\mathrm{on}\;\mathrm{the}\;\mathrm{resonance}\;\mathrm{wavelength}\;(\lambda_{B}$)
Grating pitch	$\mathrm{Determines}\;\mathrm{the}\;\mathrm{resonance}\;\mathrm{wavelength}\;(\lambda_{B}$)
Number of periods	Affects the peak reflectivity and 3 dB bandwidth [25]

**Table 3:** Comparison between the lab calibration and the embedded sensor.

	a (pm/°C)	b (nm)	$R^{2}$
Calibration	78.4	1538.1	0.948
Embedded sensor	85.8	1537.5	0.944

## Data Availability

The data that support the findings of this study are available from the corresponding author, G.S., upon reasonable request.
